# Eight Genome Sequences of Cluster BE1 Phages That Infect *Streptomyces* Species

**DOI:** 10.1128/genomeA.01146-17

**Published:** 2018-01-11

**Authors:** Lee E. Hughes, Christopher D. Shaffer, Vassie C. Ware, Issac Aguayo, Rahat M. Aziz, Swapan Bhuiyan, Isaac S. Bindert, Canyon K. Calovich-Benne, Jordyn Chapman, Richard Donegan-Quick, Amal Farooq, Cynthia Garcia, Lee H. Graham, Baylee Y. Green, Margaret A. Kenna, Emily R. Kneeream, Christian E. Laing, Catherine M. Mageeney, Sandra N. Meridew, Abigail R. Mikolon, Ryann E. Morgan, Subhayu Nayek, Idowu D. Olugbade, Kim C. Pike, Lauren E. Schlegel, Taylor C. Shishido, Tara Suresh, Nikita Suri, Kathleen Weston Hafer, Rebecca A. Garlena, Daniel A. Russell, Steven G. Cresawn, Welkin H. Pope, Deborah Jacobs-Sera, Graham F. Hatfull

**Affiliations:** a Department of Biological Sciences, University of North Texas, Denton, Texas, USA; b Department of Biology, Washington University in St. Louis, St. Louis, Missouri, USA; c Department of Biological Sciences, Lehigh University, Bethlehem, Pennsylvania, USA; d Department of Biology, Wilkes University, Wilkes-Barre, Pennsylvania, USA; e Department of Biological Sciences, University of Pittsburgh, Pittsburgh, Pennsylvania, USA; f Biology Department, James Madison University, Harrisonburg, Virginia, USA

## Abstract

Cluster BE1 *Streptomyces* bacteriophages belong to the *Siphoviridae*, with genome sizes over 130 kbp, and they contain direct terminal repeats of approximately 11 kbp. Eight newly isolated closely related cluster BE1 phages contain 43 to 48 tRNAs, one transfer-messenger RNA (tmRNA), and 216 to 236 predicted open reading frames (ORFs), but few of their genes are shared with other phages, including those infecting *Streptomyces* species.

## GENOME ANNOUNCEMENT

The increasing numbers of bacteriophages that infect *Streptomyces* hosts and have sequenced genomes available show them to have considerable genetic diversity (1), similar to that reported for phages of *Mycobacterium* (2) and *Gordonia* (3) hosts. Here, we report eight newly isolated *Streptomyces* bacteriophages recovered using either direct plating or enrichment on three *Streptomyces* hosts, *Streptomyces griseus* ATCC 10137 (phages NootNoot, Paradiddles, Samisti12, Sushi23, and Warpy), *Streptomyces lividans* JI 1326 (phages Jay2Jay and Peebs), and *Streptomyces viridochromogenes* DSM40736 (phage Mildred21). Isolates were obtained from soil samples collected in Missouri, Pennsylvania, and Texas (Table 1).

**TABLE 1:** Properties of eight *Streptomyces* phages

Phage name	Isolation host	Genome size (bp)	Terminal repeat size (bp)	No. of ORFs	No. of RNAs^a^	Origin	GenBank accession no.
Jay2Jay	*S. lividans*	133,531	10,778	235	46	Tresckow, PA	KM652554
Mildred21	*S. viridochromogenes*	131,976	10,818	234	49	St. Louis, MO	MF155946
NootNoot	*S. griseus*	131,086	10,787	221	46	Keller, TX	MF347636
Paradiddles	*S. griseus*	133,486	10,778	216	47	Denton, TX	MF347637
Peebs	*S. lividans*	133,047	11,072	226	44	Wilkes-Barre, PA	MF347638
Samisti12	*S. griseus*	133,710	10,666	226	45	Denton, TX	MF347639
Sushi23	*S. griseus*	133,917	11,074	229	45	Bethlehem, PA	MF358542
Warpy	*S. griseus*	132,996	11,458	233	45	Wrightsville, PA	MF358541

a tRNA and tmRNA genes. All eight phages contain one tmRNA gene.

Phage genomes were sequenced using the Illumina MiSeq platform (except for Samisti12, which was sequenced using Ion Torrent), and genomes were assembled using Newbler. The resulting assemblies each contained a single major contig with at least 185× coverage, long terminal repeats, and defined ends (Table 1). The physical ends of each genome were identified by a substantial increase in the number of reads beginning at a single base position and an approximately 2-fold increase in coverage between these ends defined the terminal repeats. Their genomes range in size from 131,086 bp to 133,917 bp, with a mean genome size of 132,969 bp (Table 1). All phages have G+C contents of 50% ± 0.5%, approximately 20% lower than those of their hosts. The eight phages are closely related, with pairwise average nucleotide identities from 0.79 to 0.98, and they are grouped together in subcluster BE1; Mildred21 is the least similar to other BE1 phages. Electron microscopy shows these phages to have a siphoviral morphology and isometric capsids.

All genomes were annotated using Glimmer and GeneMarkS and refined using BLAST alignments to previous manually annotated genes; start codon choice was evaluated using multiple sequence alignments of closely related genes. Putative functions were assigned using BLASTP (4) and HHpred (5) or by protein-threading assessments using Phyre2 (6); potential functions were assigned to approximately 20% of the protein-coding genes. Annotated gene functions include virion structure and assembly, DNA/RNA metabolism, lysis, and DNA packaging.

The direct terminal repeats of approximately 11 kbp are the longest found in any of the actinobacteriophages to date, and they are similar in size to previously reported long repeats in phages, such as T5 and SPO1, which have terminal repeats of 10,139 and 13,185 bp, respectively (7, 8). The direct repeats are predicted to include 20 to 25 open reading frames, including an *lsr2*-like gene; the other genes are of unknown function. These subcluster BE1 phages each carry a single transfer-messenger RNA (tmRNA) gene and the largest numbers of tRNA genes (43 to 48) among any phages of actinobacterial hosts, including at least one tRNA gene for each of the 20 amino acids. This large tRNA gene repertoire could act to counter tRNA-based degradation defense systems or to optimize gene expression in hosts that have widely different G+C contents and differing codon usage preferences (9).

### Accession number(s).

The GenBank accession numbers are shown in Table 1.
